# Integrated conjugative plasmid drives high frequency chromosomal gene transfer in *Sulfolobus islandicus*

**DOI:** 10.3389/fmicb.2023.1114574

**Published:** 2023-01-23

**Authors:** Ruben L. Sanchez-Nieves, Changyi Zhang, Rachel J. Whitaker

**Affiliations:** ^1^Carl R. Woese Institute for Genomic Biology, University of Illinois Urbana-Champaign, Urbana, IL, United States; ^2^Department of Microbiology, University of Illinois Urbana-Champaign, Urbana, IL, United States

**Keywords:** recombination, gene transfer, conjugative plasmid, *Sulfolobus islandicus*, archaea, conjugation frequency

## Abstract

Gene transfer in crenarchaea has been observed within natural and experimental populations of *Sulfolobus.* However, the molecular factors that govern how gene transfer and recombination manifest themselves in these populations is still unknown. In this study, we examine a plasmid-mediated mechanism of gene transfer in *S. islandicus* that results in localized high frequency recombination within the chromosome. Through chromosomal marker exchange assays with defined donors and recipients, we find that while bidirectional exchange occurs among all cells, those possessing the integrated conjugative plasmid, pM164, mobilize a nearby locus at a significantly higher frequency when compared to a more distal marker. We establish that *traG* is essential for this phenotype and that high frequency recombination can be replicated in transconjugants after plasmid transfer. Mapping recombinants through genomic analysis, we establish the distribution of recombinant tracts with decreasing frequency at increasing distance from pM164. We suggest the bias in transfer is a result of an Hfr (high frequency recombination)-like conjugation mechanism in this strain. In addition, we find recombinants containing distal non-selected recombination events, potentially mediated by a different host-encoded marker exchange (ME) mechanism.

## 1. Introduction

The hyperthermophilic *Sulfolobales* are the genetically tractable model system of the crenarchaea, and the model archaeon that shares a most recent common ancestor with eukaryotes. Mechanisms of genetic exchange and recombination are not well understood in the Crenarchaea; however, they are important molecular components that may hold a key to understanding the origin of meiosis and eukaryogenesis (Bernstein et al., 1985; Gross and Bhattacharya, 2010). The Sulfolobales have been shown to exchange genetic markers when complementary auxotrophic mutants are co-incubated and plated on selective media. Previous studies showed that selected markers were transferred during marker exchange (ME) at relatively equal frequencies for both auxotrophs (Grogan, 1996). Further work showed that selected marker transfer occurs in small discontinuous patches that are dependent on minimal homology (Hansen et al., 2005). In most studies, ME is up-regulated with DNA damaging agent exposure leading to the current hypothesis that ME is a mechanism for DNA repair (Schmidt et al., 1999; Ajon et al., 2011; van Wolferen et al., 2015, 2016, 2020; Feng et al., 2018; Schult et al., 2018; Sun et al., 2018). It has been experimentally shown that in *Sulfolobus acidocaldarius*, exposure to DNA damaging agents results in induced type IV pilin-mediated cell aggregation (van Wolferen et al., 2016; Sun et al., 2018). Recently, it has been shown that S-layer glycosylation patterns and a specific sequence of the UpsA proteins are responsible for species-specific aggregation and ME (van Wolferen et al., 2020). In addition, a novel DNA uptake system (Ced system) that is also UV inducible has been shown to be essential for this process (van Wolferen et al., 2015). Still, the mechanism by which DNA is moved from donor to recipient cell is unknown. It has also been hypothesized that integrated conjugative elements and their transfer systems may be responsible for chromosomal DNA mobilization (She et al., 2004; Chen et al., 2005).

Sulfolobaceae possess the only experimentally characterized conjugative plasmids in Archaea (Schleper et al., 1995; Prangishvili et al., 1998; Lipps, 2006). Most of the plasmid-containing strains are from the *S. islandicus* species (Prangishvili et al., 1998; Greve et al., 2004). Conjugative plasmids and other mobile genetic elements (MGEs) integrate at tRNA sites and can be stably maintained within the chromosome (Lipps, 2006; Cadillo-Quiroz et al., 2012). Transfer of episomal plasmids, through conjugation between *Sulfolobus* spp., can be readily achieved in the lab through co-incubation in liquid media under shaking conditions (Prangishvili et al., 1998). Conjugative plasmids in *Sulfolobus*, described as pNOB8-like plasmids, usually possess an origin of replication, a *repA* homolog (implicated in replication), *parAB* partitioning homologs, a highly conserved *plrA* gene (implicated in plasmid regulation), a site-specific integrase, and transfer gene homologs *trbE* and *traG*, among other variable open reading frames (ORFs) (Stedman et al., 2000; Greve et al., 2004; She et al., 2004). Sequence alignments between pNOB8-like transfer genes, such as *trbE* and *traG*, have also shown that these genes are among the most conserved between plasmids (She et al., 2001). pNOB8-like plasmids can sometimes produce defective smaller plasmids that lack their transfer genes and can only be maintained in the presence of an intact copy, denoting the importance of these genes for mobilization (Stedman et al., 2000). Although molecular studies into the components of *Sulfolobus* plasmid transfer machinery have not been undertaken, studies in bacterial plasmids, such as the F plasmid in *Escherichia coli* and the Ti plasmid of *Agrobacterium* spp., have shown that TraG and TrbE, respectively are type IV secretion system conjugation components which form an essential function of the mate-pair formation (mpf) machinery and thus conjugation itself (Firth and Skurray, 1992; Li et al., 1999). The F plasmid in *E. coli*, specifically, has shown the ability to recombine into the chromosome and transfer chromosomal genes in a linear fashion from the origin of transfer (Hfr) (Lloyd and Buckman, 1995).

At a population level, there is evidence for chromosomal gene exchange and episomal plasmid transfer in *Sulfolobus* spp. (Schleper et al., 1995; Cadillo-Quiroz et al., 2012). Previous studies from isolate genomes of *S. islandicus* from a single hot spring in Kamchatka, Russia, have shown that closely related groups can maintain a level of gene flow which is higher within each group than it is between them, fitting the biological species definition. There is populational genomics evidence for differences in recombination between two “species”, with the highest exchange rates among strains in the “Red” group between isolates M.16.4 and M.16.40, although analysis of proximal regions to recombinant tracts suggests this has no relationship to regional homology (Cadillo-Quiroz et al., 2012). Genome analysis shows there are differences in recombination and structural changes in certain parts of the chromosome (Krause and Whitaker, 2015). It is not known whether this chromosomal architecture is a function of mechanisms of gene transfer and recombination or a function of differences in selective forces removing or maintaining variation in different regions.

In this study we show that the presence of an integrated conjugative plasmid, pM164, increases the frequency of marker exchange near its integration site in a manner that is dependent on the plasmid encoded TraG. Because high frequency of recombination occurs surrounding the plasmid integration site, we hypothesize that an Hfr-like (high frequency recombination) mechanism led to the bias in recombination frequency near the integrated element while background levels of recombination occur in other locations.

## 2. Materials and methods

### 2.1. *Sulfolobus* strains and growth conditions

*S. islandicus* M.16.4 and its derivatives described in Table 1, were grown in tissue culture flasks (Falcon) at 73°C to 76°C without shaking. DY (dextrin-tryptone) media (pH 3.45) was used in all cases and contained the following components (per 1 L of Milli-Q H_2_O): basal salts (K_2_SO_4_, 3.0 g; NaH_2_PO_4_, 0.5 g; MgSO_4_, 0.145 g; CaCl_2_ ⋅ 2H_2_O, 0.1 g), 20 μl trace mineral stock solution (3.0% FeCl_3_, 0.5% CoCl_2_ ⋅ 6H_2_O, 0.5% MnCl_2_ ⋅ 4H_2_O, 0.5% ZnCl_2_, and 0.5% CuCl_2_ ⋅ 2H_2_O), 0.2% (wt/vol) dextrin, and 0.1% (wt/vol) tryptone. Plate media was prepared by pre-warming 2x DY media, supplemented with 20 mM of MgSO_4_ and 7 mM CaCl_2_ ⋅ H_2_O, and mixing an equal volume of a freshly boiling 1.7% (wt/vol) Gelrite solution. To grow uracil and agmatine auxotrophic strains, DY media was supplemented with a final concentration of 20 μg/ml of uracil and 50 μg/ml of agmatine. For 5-FOA counterselections, a final concentration of 50 μg/ml was also added to DY plate media. For general molecular cloning manipulations *Escherichia coli* (NEB 5-alpha competent *E. coli*) was utilized on LB media. Ampicillin (100 μg/ml) was added to media when required.

**TABLE 1:** Strains and plasmids used in this study.

Strain	Genotype/Feature	References
*S. islandicus* M.16.4 (Red group)	Wild type (*argD*^+^*pyrEF*^+^*lacS*^+^)	Reno et al., 2009 PNAS
*S. islandicus* M.16.40 (Red group)	Wild type (*argD*^+^*pyrEF*^+^*lacS*^+^)	Cadillo-Quiroz et al., 2012
*S. islandicus* M.16.2 (Red group)	Wild type (*argD*^+^*pyrEF*^+^*lacS*^+^)	Cadillo-Quiroz et al., 2012
*S. islandicus* M.16.27 (Blue Group)	Wild type (*argD*^+^*pyrEF*^+^*lacS*^+^)	Reno et al., 2009 PNAS
RJW004	M.16.4Δ*argDΔpyrEFΔlacS*	Zhang et al., 2013a
Δ*traG-*RJW004	Δ*argD::argDΔpyrEF::pyrEFΔlacS*Δ*traG*	This Study
RJW007	M.16.4Δ*argDΔpyrEFlacS^+^*	Zhang and Whitaker, 2018
*S. islandicus* M.16.2p	M.16.2 harboring conjugative plasmid pM164	This study
**Plasmid**		
pSe-RP-*StoargD-traG*	pSeSd-StoargD with *S. islandicus* M.16.4 CRISPR repeats and *traG* spacer + HR template; created through NEB Hifi assembly	This Study

### 2.2. Mating assay

**FIGURE 1:**
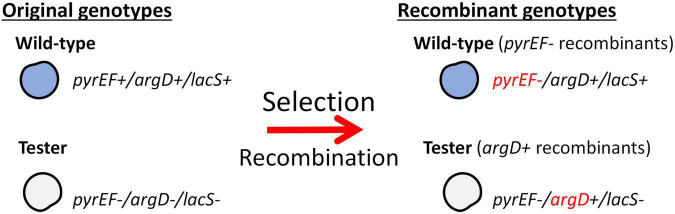
Selection of *argD*+ *pyrEF*- recombinant genotypes. This schematic shows necessary genotypes utilized in the mating assays and the recombinants produced through selection. Selection is based on agmatine prototrophy and resistance to 5-FOA.

The mating assays for *S. islandicus* strains were performed through selection of agmatine prototrophy and 5-FOA resistance. Two genotypes are needed in order to perform the mating assay: a strain with functional *lacS, argD*, and *pyrEF* loci, and a strain with deletions in these three markers (Figure 1).

In all mating assays, both partner strains were grown to a mid-log phase, as detailed above, in either DY media or DY media supplemented with uracil and agmatine. Once strains reached their mid-log phase, their optical density (600 nm) was measured and equalized using warm DY media to an OD_600_ of 0.3. The two strains were then mixed in equal parts and incubated at room temperature for 15 min. The co-incubates were then incubated under shaking conditions (180 rpm) at 76°C overnight. Following incubation, the mixed cultures were washed to remove agmatine using centrifugation and equalized to an OD_600_ of 0.5. Finally, cultures underwent serial dilutions and were plated in either selective DY media containing 5-FOA and uracil (10^0^ and 10^–1^ dilutions) or non-selective DY media containing agmatine and uracil (10^–4^ and 10^–5^ dilutions). After cells are plated, plates are incubated for 7–14 days at 76°C. Once colonies are observed on plates, an X-gal solution (2 mg/ml) was sprayed on both selective and control plates to determine original background. After the genotype is confirmed through PCR, the colonies are counted and analyzed to determine the recombination frequency of both the tester strain and either the wild-type or mutants with all three genetic markers.

Recombinant frequencies are calculated through the following formula: $recombinantfrequency=\frac{recombinantsG1}{controlG1}$, where *recombinantsG1* represents the cfu/ml of colonies on selective media of a particular genotype. *ControlG1* represents the cfu/ml of colonies counted on non-selective media of the same genotype, G1. Colonies are verified through PCR amplification using primers: *argD_chk-F/R* & *M16_pyrEFII-F/R* (Supplementary Table 1).

### 2.3. Construction of *traG* in-frame deletion plasmids

Gene knockout shuttle vectors were constructed using NEBuilder Hifi assembly kit (NEB, USA) and according to manufacturer’s protocol. IDT G-Blocks were synthesized for the *traG* spacer and recombination template. The shuttle vectors were cloned in NEB 5-alpha competent *E. coli* (High Efficiency; NEB, USA) cells and plated on LB with ampicillin (100 μg/ml). Shuttle vectors were subsequently isolated using the QIAprep Spin Miniprep Kit (Qiagen, USA) as per manufacturer’s instructions.

### 2.4. Genetic manipulation of *S. islandicus* strains

Once knockout plasmids were isolated, plasmids were electroporated into competent RJW004 cells and incubated in incubation buffer for 1 hr (Zhang et al., 2013a). Transformations were then plated onto DY plates and incubated for 10–14 days. After colonies appeared on media, X-gal staining and PCR amplification was performed on colonies to confirm vector presence in the strain. Once colonies were confirmed, they were grown in liquid DY media to mid-log phase.

These strains were subsequently plated onto counter-selective DY media containing uracil, agmatine, and 5-FOA at concentrations mentioned above. Plates were incubated for 10–14 days. After incubation, colonies were stained with X-gal and picked for PCR amplification of target genes. Subsequently verified colonies were streaked for purification and subsequent DNA extraction and whole-genome sequencing.

Further manipulation of RJW004-Δ*traG* was needed to obtain the proper genotype for mating assays (Figure 4). The *argD, pyrE*, and *pyrF* genes were inserted through purification and electroporation of M.16.4-derived amplicons (Zhang and Whitaker, 2018). Because the source is isogenic to RJW004, no homology modifications were introduced into the amplification of the genes. Enrichment for 14 days was necessary for efficient isolation of *pyrEF*^+^ colonies. Genomic DNA was extracted and sent to sequencing for further verification.

**FIGURE 2:**
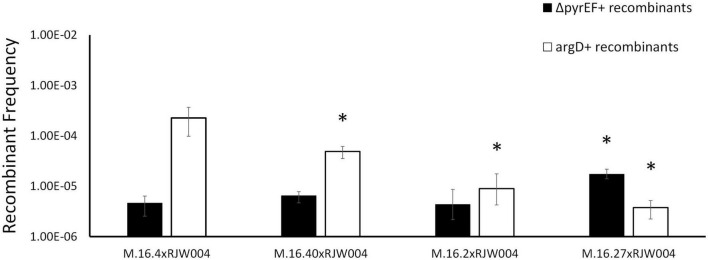
Recombination frequencies of Red species isolates and Blue species representative (M.16.27) in mating assays with the tester strain, RJW004. White bars represent the recombinant frequency of *argD* recombinants, while black bars represent the recombinant frequency of Δ*pyrEF* recombinants. Error bars represent highest and lowest replicate for each assay, *represents a *p*-value of <0.05 when compared to M.16.4 cross.

**FIGURE 3:**
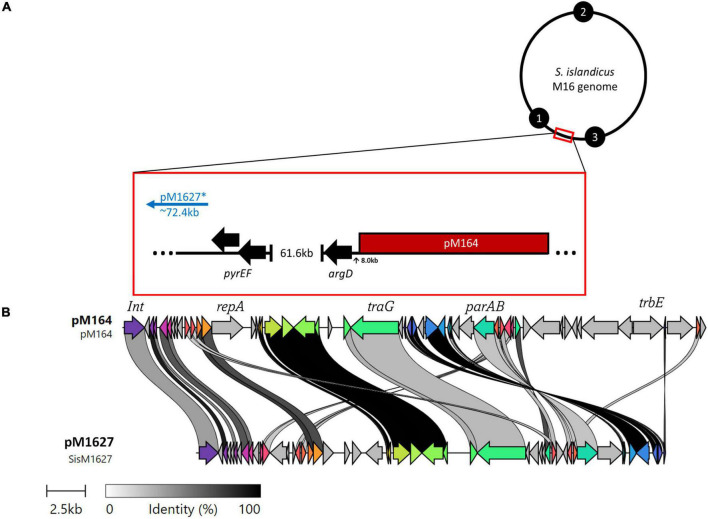
**(A)** M16 genomes possess broad similarities, thus the genomic position of pM164 and pM1627 (*not a complete plasmid) can be resolved by a general model in reference to the selective markers *argD* and *pyrEF*. Black numbered circles represent replication origins. **(B)** A clinker nucleotide alignment of pM164 and pM1627, which is missing essential plasmid components such as *repA, parA*, and *trbE.*

**FIGURE 4:**
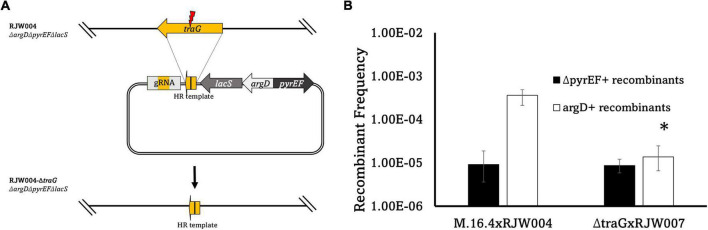
**(A)** Schematic diagram of *traG* gene deletion based on CRISPR-Cas system in *S. islandicus* M.16.4. The genome editing plasmid was designed by taking advantage of the native CRISPR-Cas system in *S. islandicus* to build a gRNA targeting *traG*. To avoid marker recombination, argD from *S. tokodaii* and *pyrEF*/*lacS* from *S. solfataricus* were utilized instead on native markers. **(B)** Recombinant frequencies of the *traG* deletion mutant compared to wild type. Error bars represent highest and lowest replicate for each assay, *represents a *p*-value of <0.05 when comparing to M.16.4 argD frequency.

### 2.5. Conjugation of pM164

Conjugation of pM164 into *S. islandicus* M.16.2 was performed according to Prangishvili et al. (1998) with minor modifications and a selection scheme (Supplementary Figure 1). In general, *S. islandicus* RJW004 was co-incubated with *S. islandicus* M.16.2 at a donor: recipient ratio of 1:10,000 for 48 h under shaking conditions in liquid DY media containing uracil and agmatine at 76°C. After incubation, the co-culture was diluted using serial dilutions to 10^–3^ of the original incubation and plated on DY media to select against RJW004. After incubation for 10 days, colonies were picked and verified for pM164 in the chromosome and original background through PCR amplification of pM164 region and the strain verification, respectively. M.16.2p was then purified through streaking and sent to sequencing using Illumina short reads. Additionally, circularization of pM164 was assessed through amplification of plasmid distal regions (Supplementary Figure 3).

### 2.6. Sequencing SNP analysis and bioinformatics

SNP analyses were performed on recombinant isolates from three independent tester/M.16.2p mating assays. Colonies were isolated and transferred to liquid DY media until mid-log phase. Subsequently, DNA extraction and Illumina short read sequencing were performed on 30 RJW004 recombinants. Sequencing files were then analyzed for M.16.2p-specific donor SNPs through Breseq analysis (Deatherage and Barrick, 2014). SNPs were then mapped onto an M.16.4 reference for analysis. Alignment for Figure 3 was performed using Clinker (Gilchrist and Chooi, 2021). Variant positions in core genome were assessed using Spine and Nucmer (Kurtz et al., 2004; Ozer et al., 2014).

### 2.7. Statistical analysis

Statistical analysis was performed on mating assays in all cases. Comparisons were done utilizing ANOVA when experiments involved more than two assays. In cases where two assays were compared a *t*-test was performed to assess significant differences. In all cases *p*-value-0.05 is utilized.

## 3. Results

### 3.1. Mutnovsky Red group show varying levels of recombination frequencies

The natural isolates utilized in this study were isolated from the Mutnovsky region in Kamchatka Russia in 2010. We focus on one of two closely related but distinct groups (the Red group) observed to be diverging over time in a manner that represents incipient speciation from within the same hot spring (Cadillo-Quiroz et al., 2012). The Red group is composed of three natural isolates which are closely related and have shown evidence of high levels of recombination between them. At the nucleotide level, the Red group possess 6,052 variant positions across core genome and an ANI (average nucleotide identity) of above 99.75% in all cases. We include one Blue group strain, M.16.27, for comparison of transfer among incipient species.

To test recombination frequencies between these strains we utilized a tester strain, RJW004, which is an M.16.4 background strain possessing three gene deletions (Δ*argD, ΔpyrEF*, and Δ*lacS*). This strain is crossed with wild-type strains in mating assays described in Figure 1 (Zhang et al., 2013a,b). The *lacS* gene is not selected in this assay but used to differentiate donor and recipient genome backgrounds on selective plates through X-gal staining. Selection on uracil/5-FOA (5-Fluoroorotic acid) containing plates without agmatine selects for strains in wild type background (blue) that receive the Δ*pyrEF* deletion locus from the tester (*argD^+^pyrEF^–^lacS^+^*), and the tester strain (white) that receives the *argD* locus from the WT (argD^+^pyrEF^–^lacS^–^) (Figure 1). In the M.16.4 strain the selected markers (Δ*pyrEF, argD*) are separated by 62Kb. To differentiate deletion from spontaneous mutations in each experiment WT and tester strains are plated on selective plates and the number of spontaneous mutations is subtracted from total.

Figure 1 shows bi-directional recombination when the tester strain is crossed with WT strains from the same population. Figure 2 shows the recombination profiles of the above-mentioned natural isolates for our two selective markers. The recombination frequency of the Δ*pyrEF* locus from the tester to WT strains is consistent, independent of the WT background, yet slightly higher in the cross with M.16.27. In contrast, *argD*^+^ recombinants showed substantial variation with M.16.4 having the highest recombination frequency, M.16.27 having the lowest frequency, and the M.16.2 assay possessing the lowest donor frequency of the red isolates (Figure 2).

### 3.2. pM164 conjugation and transfer play a role in high frequency recombination

Interestingly, an integrated conjugative plasmid, named pM164, was found 8086bp from the highly transferred *argD* marker in M.16.4 and M.16.40 (Figure 3). PCR amplification has also shown evidence of excision in M.16.4 along with the integrated version, providing evidence that pM164 is an active pNOB8-like element. Because the *argD* recombination frequency was associated with pM164, in both M.16.4 and M.16.40, we hypothesized that pM164 may well be driving the transfer of proximal genes like *argD*. We note that M.16.27 has a divergent plasmid integrated in a different position relative to markers and relatively closer (∼72 kb) to *pyrEF* than to *arg*D. This plasmid, however, lacks important plasmid components such as *repA, parA*, and *trbE.*

In bacterial conjugation models *traG* (*M164_1618*), encoding for a VirD4-type conjugation component, is essential for plasmid transfer as it mediates the interactions between the mating pore and the DNA transfer system (Arends et al., 2013; Kohler et al., 2013). The *traG* gene was identified through comparisons to pNOB8 and its genomic characterization (She et al., 1998). TraG in *Sulfolobus* spp. contains a conjugation-specific type IV secretion system (T4SS) domain found in bacterial conjugation proteins such as VirD4, TrwB, and TraG (She et al., 1998). When compared, three motifs are present that correspond to bacterial conjugation proteins such as TrwB in *E. coli* and with the archaeal HerA helicase (Supplementary Figure 2). Thus, we hypothesized that a *traG* deletion in pM164 would abrogate transfer and thus the associated high frequency recombination of *argD*. We created a marker-less in-frame deletion of the pM164 *traG* (M164_1618) in RJW004, along with the appropriate marker insertions, to create an *argD^+^pyrEF^+^lacS^–^traG^–^* strain by using the endogenous CRISPR-Cas system-based genome editing (Li et al., 2015). Because this strain is *lacS^–^*, it was paired with RJW007, an *argD^–^pyrEF^–^lacS^+^* tester, to yield selectable recombinant genotypes (Table 1). An M.16.4 (wt)/RJW004 (tester) mating assay was performed for comparison. We found a significant decrease in *argD* recombination frequencies when *traG* was not present in the *argD* donor strain (Figure 4). Background levels of marker exchange were maintained in these crosses, however, the bias for the *argD* marker was absent.

### 3.3. pM164 transfer increases recombination frequency in transconjugants

We tested whether pM164 is mobilizable and can independently transfer its high recombination phenotype to a naïve strain. The red isolate, M.16.2, did not possess a conjugative plasmid but has an identical *att* site and selective marker locations, as such we chose to conjugate pM164 into M.16.2. The conjugation protocol was adapted from Prangishvili et al. (1998) and is diagramed in Supplementary Figure 1 (see “3. Materials and methods”). First, Δ*traG*-RJW004 was utilized as a pM164 donor to test the importance of *traG* to plasmid transfer in *S. islandicus* and found that only 1/99 recipient cells possessed pM164. Thus, much like in bacteria, *traG* is essential for conjugation in *S. islandicus* (Waters et al., 1992).

Using *traG+* donor we were able to obtain M.16.2 transconjugants possessing pM164. Transconjugants were verified for strain identity and the appropriate pM164 insertion event using PCR amplification of the *att* site (Supplementary Figure 1). 99% of colonies surveyed possessed pM164 in the insertion site, showing strong evidence of insertion site specificity and high efficiency of transfer (She et al., 2004). Highly efficient transfer is characteristic of conjugative plasmids in *Sulfolobus*, as has been observed previously by Prangishvili et al. (1998). Of the colonies verified to possess pM164, three were purified and sent for sequencing to further confirm the strain and the presence of pM164 in the appropriate locus.

**FIGURE 5:**
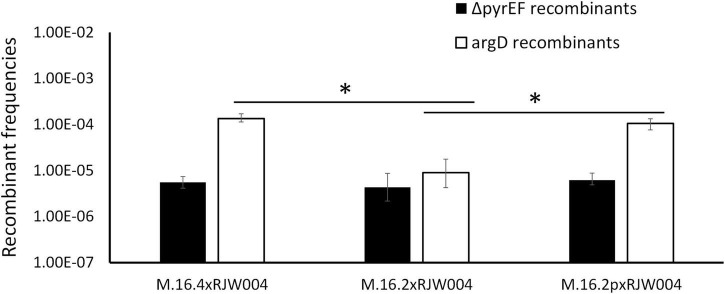
Recombination frequencies of genetic markers in M.16.2 transconjugant mating assays compared to parent strain. White bars represent the recombinant frequency of *argD*, while black bars represent the recombinant frequency of Δ*pyrEF*. Error bars represent highest and lowest replicate for each assay, *represents a *p*-value of <0.05 when comparing to M.16.2 *argD* frequency.

We mated M.16.2p, a pM164 transconjugant of the parental strain M.16.2, with the naive strain to assess the recombination frequencies of the two selectable markers (Figure 5). Through sequencing we confirmed that M.16.2p does not possess any other MGE other than pM164 and further confirmed that pM164 is integrated into the correct position. We show that high frequency recombination of *argD* follows insertion of pM164 into the appropriate tRNA*-*adjacent attachment site. Thus, we show direct evidence of an integrated plasmid influencing core genome recombination dynamics in Archaea. These results provide evidence that indeed pM164 and not the strain is the deciding factor in localized high frequency recombination of *argD.*

### 3.4. Transferred recombinant tracts suggest transfer from plasmid is unidirectional in a background of unselected recombinant tracts

**FIGURE 6:**
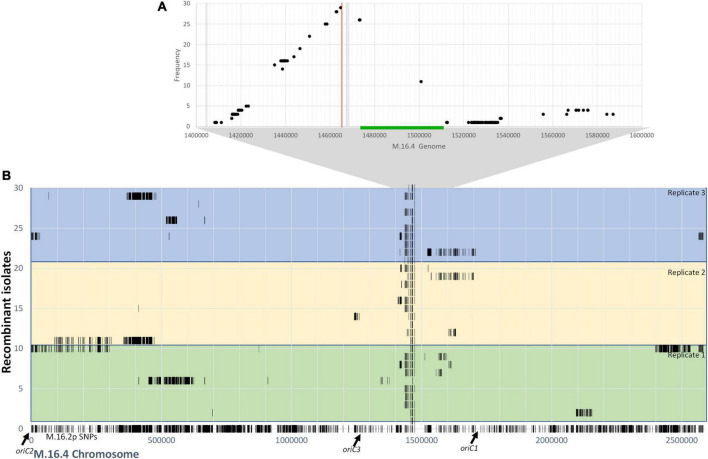
**(A)** Frequency of M.16.2p donor SNPs within the *argD*/pM164 region in RJW004 recombinants. The green bar represents pM164 and the red line represents the *argD* locus. Gray bar represents variable gene content between strains in M.16.2p. **(B)** Donor *SNP distribution along the M.16.4 chromosome in RJW004 recombinants.* The bottom (line 0) represents the SNP positions of M.16.2p (donor) mapped on to M.16.4 genome. The colored blocks represent each replicate, each containing 10 independent isolates.

To gain further understanding into the mechanism of pM164 transfer, we sequenced 10 RJW004 recombinants from three independent M.16.2p mating assays for a total of 30 isolates. Each isolate represents independent recombination events within each experiment. We assess chromosomal regions that are transferred from M.16.2p to our tester strain, RJW004 (Figure 6A). The M.16.2p donor locus that stretches between *argD* and the pM164 integration site were differentiated from the recipient (RJW004) locus through SNP analysis (Figure 6). Because M.16.2 is highly similar to M.16.4, SNP positions scattered throughout the chromosome can be utilized as markers for recombination. We find consistent recombination tracts leading from the selected marker *argD* to pM164 in an uninterrupted manner. We also find a slope in recombinant SNPs upstream of *argD* that is not observed on the other end of pM164. From these data we infer that pM164 transfers unidirectionally while integrated in the chromosome, in a manner similar to Hfr strains in *E. coli.* In addition, we also found that 17/30 isolates possess unselected recombination events throughout the chromosome.

We find that long unselected donor tracts do not correlate with genomic distance from pM164. Thus, we hypothesize that although pM164 has a localized effect made apparent by the high frequency of unselected donor SNPs surrounding the *argD/att* region, the ME/Ced system most likely accounts for most of the intercellular mobilization of DNA throughout the chromosome. The localized high frequency effect is most likely due to unidirectional integrated plasmid transfer from a plasmid origin toward *argD*, these tracts are then resolved through homologous recombination between the chromosomes, defining the recombination tract lengths. These data, along with the essentiality of plasmid transfer through a TraG-mediated mechanism, show that in *S. islandicus* integrated plasmids can consistently and reproducibly create high-frequency marker exchange in nearby chromosomal loci.

## 4. Discussion

This study has uncovered evidence of plasmid-mediated high-frequency transfer of DNA in the strain M.16.4 from Kamchatka, Russia. We show that high frequency transfer requires TraG (Figure 4) and is transferable through plasmid conjugation to M.16.2, demonstrating that this mechanism depends upon the plasmid pM164. Genome sequencing of recombinants showed that tracts within the *argD* genomic context mostly extend through to the pM164 *att* site which we interpret as integrated plasmid transfer in a manner similar to Hfr. This process increases recombination frequencies unidirectionally in this region directly adjacent to *argD*. This study is the first to directly demonstrate a role for integrated plasmids in the mobilization of chromosomal loci in *Sulfolobus.*

In this work the ME system is active under uninduced conditions, which allows for genetic selections of chromosomal markers through co-incubation and plating on selective media. This was first noted by Grogan (1996) and has been the basis for many studies including those involved in the recombination mechanics with *Sulfolobus* and the UV-up-regulated system that allows for lateral gene transfer (Grogan, 1996; Reilly and Grogan, 2002; Hansen et al., 2005; Ajon et al., 2011; Rockwood et al., 2013; Zhang et al., 2013b; van Wolferen et al., 2020, 2016). Here, we find an association of a 37 kb integrated conjugative plasmid and a highly recombining marker located 8 kb from the integration site. Interestingly, Cadillo-Quiroz et al. (2012) found that M.16.4 and M.16.40 (both contain pM164 variants) are strong DNA donors when compared to M.16.2, which does not possess an integrated plasmid. Furthermore M.16.27, from a co-existing, closely related species, contains a plasmid that does not confer this phenotype (Figure 2). Our findings, in conjunction with previous studies looking into defective plasmids which lose *traG*, have shown that the protein encoded by *traG* plays a major role in archaeal plasmid transfer and is essential for this mechanism, although further molecular studies on the transfer mechanism of *Sulfolobus* spp. plasmids are lacking (Stedman et al., 2000). Specifically, genetic and biochemical analyses are needed to decipher more of the transfer components and their respective functions. Both our recombination assay with the natural isolate M.16.2 (Figure 2), RJW004Δ*traG* (Figure 4) and previous work in *S. acidocaldarius* show bidirectional recombination in the absence of an active plasmid, suggesting an additional host-encoded mechanism for DNA transport (Grogan, 1996; van Wolferen et al., 2016).

In *Sulfolobus* plasmids, *traG* is one of the only discernible transfer genes that can be identified through annotation as conjugation-specific proteins (the other being *trbE*). It is currently not known whether *traG* plays a similar role in *Sulfolobus* as it does in bacteria, as the molecular components of the *Sulfolobus* plasmid transfer apparatus have not been subjected to genetic or biochemical analysis in the way their bacterial counterparts have. The structure of TraG in *Sulfolobus*, however, possess similar motifs to bacterial T4SS conjugation components, such as *E coli* TrwB and other *Sulfolobus* pNOB8-like plasmids (Supplementary Figure 2; She et al., 1998).

Through donor SNP analysis of *argD+* recombinant isolates we find that this high frequency phenomenon produces, in most cases, uninterrupted donor SNPs in the area between *argD*, which is under selection, and pM164, which is not (Figure 6). These results suggest that the high frequency recombination observed in *argD* is due to the co-transfer of chromosomal DNA through a TraG-mediated mechanism that is then recombined into the recipient. Because of the slope observed at only one side of pM164 and not the other, our current model is that high recombination of *argD* is driven by unidirectional transfer of pM164 in a manner similar to Hfr strains in *E. coli.* These recombination tracts, spanning at least 8 kb, are in contrast to previous recombination dynamics observed in *S. acidocaldarius* that show minimal recombination past 300 bp of distance between selective markers, showing evidence of differing mechanisms present in this study (Hansen et al., 2005).

We hypothesized that an Hfr-like mechanism through a plasmid may explain this phenomenon. Recombination frequencies for the *pyrEF* locus are in all cases ∼10^–5^ as is also shown in for the *argD* marker when pM164 is not present (Figures 2, 5). Interestingly, this frequency is similar to the recombination frequency observed by Ajon et al. (2011) and Hansen et al. (2005) in *S. acidocaldarius* in induced condition. Here we find higher frequencies (∼10^–3^) for *argD* in uninduced conditions. Similar phenomena, involving chromosomal high recombination near a mobile genetic element, have been observed in *Staphylococcus aureus* but over a much smaller scale and involving a conjugative transposon that circularizes with proximal parts of the host chromosome (Everitt et al., 2014). In this alternative model, plasmid DNA is excised imprecisely, taking flanking chromosomal regions with it, which then transfer through the plasmid-encoded transfer system and the genes in its chromosomal vicinity. Evidence of plasmid circularization has been noted in M.16.4 through plasmid end junction (Supplementary Figure 3). However, circularization would most likely encompass both distal regions of the inserted plasmid and not the unidirectional profile we find here (Figure 6). Thus, we find that two alternative but not mutually exclusive mechanisms involving plasmid transfer could be affecting chromosomal regions nearby such as *argD.*

Because recombination is occurring throughout the chromosome and the high sequence identity between isolates, it is difficult to observe the full effects that plasmid-mediated recombination has past the marker under selection. We found that SNPs were distributed mostly in two areas: (1) consistent tracts in the *argD-*pM164 region and (2) non-selected tracts that vary greatly in size and genomic location (Figure 6). These non-selected tracts although variable in length and continuous, in most cases, are not linked to the plasmid regions and are thus attributed to the broader ME/Ced system described by Grogan (1996) and van Wolferen et al. (2016). The ME/Ced system has been demonstrated to be a UV inducible gene import system in *Sulfolobus* spp. that do not carry a pM164-like plasmid (Grogan, 1996; Ajon et al., 2011). Whether there is a compatible donor mechanism (plasmid or otherwise) is not known. Although it has not been tested explicitly, transfer of chromosomal DNA through the Ced system has been hypothesized to be responsible for unselected ME observed genetically (Hansen et al., 2005). The Ced system is present in *S. islandicus*, therefore, if the Ced mediated uptake of chromosomal DNA is correct, we suggest that the transfer of unselected tracts in our study may be happening through this alternate system. However, from the current set of experiments, we cannot exclude the possibility that the entire chromosome is transferred through an Hfr-like conjugation mechanism then recombined in patches outside of the selected region. The potential existence of multiple systems together implies that in nature portions of the chromosome, due to proximity to an integrated plasmid, will evolve differently and potentially more rapidly than others. Conjugative plasmids in *Sulfolobus* spp. are known to transfer between species, thus this mechanism lowers the barrier for DNA to be introduced in a population (Prangishvili et al., 1998). This is in contrast to the ME/Ced system which is known to be species-specific due surface layer interactions (van Wolferen et al., 2020). Our findings show the effects of MGEs on M.16-type chromosome recombination dynamics, which were first observed in nature and then in the lab (Cadillo-Quiroz et al., 2012). We find that the transfer of pM164, an integrated conjugative plasmid, causes high frequency recombination of nearby chromosomal regions. How chromosomal DNA travels between cells is still largely unknown. In order to truly understand the effects of MGEs on chromosome dynamics more studies are needed to further elucidate the mechanism by which DNA is transferred in *Sulfolobus* spp. more generally. Particularly, more genetic and biochemical approaches are needed to both understand archaeal biology in reference to MGEs and their cell biology as a whole. This study provides evidence that a relationship between mechanisms exists, and that this relationship has evolutionary implications within populations of *S. islandicus*.

## Data availability statement

The datasets presented in this study can be found in online repositories. The names of the repository/repositories and accession number(s) can be found below: https://www.ncbi.nlm.nih.gov/bioproject/PRJNA907032.

## Author contributions

RS-N and RW designed the research and drafted the manuscript. RS-N conducted experiments and performed data analysis and completed mutant construction and evaluation. CZ contributed to the experiment design and provided published strains. RS-N, CZ, and RW contributed to revising the manuscript. All authors made fundamental contributions to the manuscript and approved the final manuscript.
