# Supplementary material for: Integrated conjugative plasmid drives high frequency chromosomal gene transfer in Sulfolobus islandicus
Source: Front Microbiol. 2023 Jan 23;14:1114574. doi: 10.3389/fmicb.2023.1114574 (PMC9899855; doi:10.3389/fmicb.2023.1114574)
Supplement: Supplementary file 1 [file Data_Sheet_1.docx]

**Supplemental Table 1** Primers used in this study

| Primer | Sequence (5’→3’) |
| --- | --- |
| argD_chk-F  argD_chk-R | ccaagacttctcacaacacc  tcaaaatggttccaatcccgatac |
| M16_pyrEFII-F  M16_pyrEFII-R | tctcgtctccctcgagtattatatatgatccatcactaagtactatgttcataaattcc  cattacaatacaatttgagtaattgttcaacatcctaatggtatcagctagattttcac |
| Int_Plasmid-F  Int_Plasmid-R | ttttatgaggacgtgccgcggtg  ttgccctggagccattctccc |
| Sel_Mar_fwd  Sel_Mar_rev | gcggccgctcctcttattattag  cgaaaagtgccacctgacctggcgaaagggggatgtg |
| pZC1_fwd  pZC1_rev | catccccctttcgccaggtcaggtggcacttttcgggg  acatgcatgcagcacaattg |
| 162_ver-Fwd  162_ver-Rev | gtttattgttatcatatctgtaaatgttgttattagcgtatatg  ccttctccgttatccaaaaggagag |
| Closed_pm164_fwd  Closed_pm164_rev | ccaagttgccacattaattgc  attacgaaatagtgctgctgg |

**Supplemental Table 2-** (See supplemental spreadsheet) This table shows the SNP profile for all recombinant isolates and the comparison of these to the background strain, RJW004 and the donor strain M.16.2p.

**Supplemental Figure 1** **a)** Conjugation and isolation of M.16.2 transconjugants through co-incubation and subsequent plating on M.16.2 selective media. **b)** colonies were verified through PCR amplification of a variable position between i) M.16.4, ii) M.16.2, and the iii) M.16.2 transconjugants for strain verification along with amplification of the expected plasmid/chromosome junction site. **c)** Isolates chosen for downstream experiments were sequenced to further confirm identity.

**Supplemental Figure 2** This partial alignment compares the M.16.4 and M.16.27 TraG to the following TraG genes found in other *Sulfolobus* plasmids: pNOB8 (NC_006493.1) and pING1 (NC_004852.1). The alignment also includes HerA from M.16.4 (ACR42286.1) and bacterial conjugation proteins from: *Bacteroides uniformis* (ABP57300.1)*, Geobacter_sulfurreducens* (NP_953171)*, Streptococcus pneumoniae* (QPN87076.1)*,* and *E. coli* (PDB: 1GKI_A). This alignment and the motifs present here are based on a previous protein alignment produced by She et al, 1998.

**Supplemental Figure 3 a)** This figure shows a schematic of the primers (black arrows) annealing to provide evidence of plasmid circularization. By amplifying outward from an integrated element, amplification can only occur after the plasmid had circularized and thus excised. **b)** The electrophoresis gel shows evidence of plasmid circularization through the method expressed in part a. We find that M.16.4 and M.16.2p, both of which possess pM164, show evidence of plasmid excision.
